# The finding and researching algorithm for potentially oscillating enzymatic systems

**DOI:** 10.18699/VJ21.035

**Published:** 2021-05

**Authors:** T.N. Lakhova, F.V. Kazantsev, S.A. Lashin, Yu.G. Matushkin

**Affiliations:** Kurchatov Genomics Center of ICG SB RAS, Novosibirsk, Russia; Kurchatov Genomics Center of ICG SB RAS, Novosibirsk, Russia; Kurchatov Genomics Center of ICG SB RAS, Novosibirsk, Russia Novosibirsk State University, Novosibirsk, Russia; Institute of Cytology and Genetics of the Siberian Branch of the Russian Academy of Sciences, Novosibirsk, Russia Novosibirsk State University, Novosibirsk, Russia

**Keywords:** oscillations, feedback, cyclic processes, modelling of biological systems, осцилляции, обратная связь, циклические процессы, моделирование биологических систем

## Abstract

Many processes in living organisms are subject to periodic oscillations at different hierarchical levels of their organization: from molecular-genetic to population and ecological. Oscillatory processes are responsible for cell cycles in both prokaryotes and eukaryotes, for circadian rhythms, for synchronous coupling of respiration with cardiac contractions, etc. Fluctuations in the numbers of organisms in natural populations can be caused by the populations’ own properties, their age structure, and ecological relationships with other species. Along with experimental approaches, mathematical and computer modeling is widely used to study oscillating biological systems. This paper presents classical mathematical models that describe oscillatory behavior in biological systems. Methods for the search for oscillatory molecular-genetic systems are presented by the example of their special case – oscillatory enzymatic systems. Factors influencing the cyclic dynamics in living systems, typical not only of the molecular-genetic level, but of higher levels of organization as well, are considered. Application of different ways to describe gene networks for modeling oscillatory molecular-genetic systems is considered, where the most important factor for the emergence of cyclic behavior is the presence of feedback. Techniques for finding potentially oscillatory enzymatic systems are presented. Using the method described in the article, we present and analyze, in a step-by-step manner, first the structural models (graphs) of gene networks and then the reconstruction of the mathematical models and computational experiments with them. Structural models are ideally suited for the tasks of an automatic search for potential oscillating contours (linked subgraphs), whose structure can correspond to the mathematical model of the molecular-genetic system that demonstrates oscillatory behavior in dynamics. At the same time, it is the numerical study of mathematical models for the selected contours that makes it possible to confirm the presence of stable limit cycles in them. As an example of application of the technology, a network of 300 metabolic reactions of the bacterium Escherichia coli was analyzed using mathematical and computer modeling tools. In particular, oscillatory behavior was shown for a loop whose reactions are part of the tryptophan biosynthesis pathway

## Abstract (ru)

Многие процессы в живых организмах подвержены периодическим колебаниям на различных иерархических уровнях их организации: от молекулярного-генетического до популяционного и экологического. Осциллирующие процессы отвечают за клеточные циклы как у прокариот, так и у эукариот, за циркадные ритмы, синхронную связь дыхания с сердечными сокращениями и др. Колебания численностей организмов в природных популяциях могут быть обусловлены собственными свойствами популяций, их возрастной структурой, а также экологическими взаимоотношениями с другими видами. Наряду с экспериментальными подходами, для исследования осциллирующих биологических систем широко применяется математическое и компьютерное моделирование. В данной статье представлены классические математические модели, которые описывают осциллирующее поведение в биологических системах. Приведены методы поиска осциллирующих молекулярно-генетических систем на примере их частного случая – осциллирующих ферментативных систем. Рассмотрены факторы, влияющие на циклическую динамику в живых системах, характерные не только для молекулярно-генетического уровня, но и для более высоких уровней организации. Обсуждается применение различных способов описания генных сетей для моделирования осциллирующих молекулярно-генетических систем, где важнейшим фактором возникновения циклического поведения является наличие обратных связей. Представлены технологии поиска потенциально осциллирующих ферментативных систем. С помощью метода, описанного в статье, проводится поэтапный процесс построения и анализа сначала структурных моделей (графов) генных сетей, а затем реконструкции математических моделей и вычислительных экспериментов с ними. Структурные модели идеально подходят для задач автоматического поиска потенциальных осциллирующих контуров (связных подграфов), структура которых может соответствовать математической модели молекулярно-генетической системы, демонстрирующей осциллирующее поведение в динамике. При этом именно численное исследование математических моделей для отобранных контуров позволяет подтвердить наличие в них устойчивых предельных циклов. В качестве примера применения технологии проанализирована сеть из 300 метаболических реакций бактерии Escherichia coli с использованием инструментов математического и компьютерного моделирования. В частности, показано осциллирующее поведение для контура, реакции которого входят в путь биосинтеза триптофана

## Introduction

Many processes in living organisms are subject to periodic oscillations at different hierarchical levels of their organization: from the molecular-genetic to the population and ecological levels. For example, at the molecular-genetic level, there are oscillations in the concentrations of p53, a protein involved in apoptosis or cell cycle delay in DNA damage, and its inhibitor Mdm2 (Prives, 1998; Lahav et al., 2004). There are also fluctuations in concentrations of hormones in the cell, such as melatonin (Boccalandro et al., 2011), prolactin, total cholesterol (Garde et al., 2000), etc.; concentrations of low molecular weight compounds, such as intracellular and intercellular calcium ion concentrations, can also oscillate (Pasti et al., 1997; Allen et al., 2000)

One well-known example of organism-wide periodic processes is circadian rhythms, for the work on which the 2017 Nobel Prize in Physiology or Medicine was awarded (Young et al., 1984; Siwicki et al., 1988; Hardin et al., 1990; Price et al., 1998). Jeffrey C. Hall, Michael Rosbash, and Michael W. Young discovered the period gene in Drosophila melanogaster, which is regulated through feedback by the PER protein underlying circadian rhythm.

In the article (Podkolodnyy et al., 2017), the authors considered genes located in liver and kidney cells that are overexpressed with a certain periodicity during the 24-hour cycle. In a subsequent paper, the authors provided an overview of various mathematical models used to model the autonomous circadian clock in mammalian cells (Podkolodnaya et al., 2017).

At the cellular level, cyclic processes can include cell cycles in both prokaryotes and eukaryotes (Cooper, 1991). Such important cyclic processes as heartbeat (Ashkenazy et al., 2001), respiration, as well as synchronous relationship between respiration and heartbeat (Yasuma, Hayano, 2004), photosynthesis (Holtum, Winter, 2003) and other similar processes occur at the level of an individual organ or functional systems of an organism.

Population waves (Chetverikov, 2009) are a classic example of cyclic processes at the population level of organization. Fluctuations in the number of organisms in natural populations can be caused both by external environmental factors and by the population’s own properties, its age structure, and ecological relationships with other species. A natural factor such as seasonal periodicity plays an important role in the cyclic processes of the population level, influencing the migration of birds, falling into anabiosis, the appearance and fall of leaves, etc.

Thus, in the article (Erdakov, Moroldoev, 2017), the authors considered the cyclicity in the population dynamics of the red vole, which varies depending on the geographical habitat and external conditions in the area. And in the paper (Pertsev, Loginov, 2011), using a stochastic model, the authors considered how the population size changes when harmful food resources are consumed. The investigation of population dynamics, often cyclical, is one of the most studied processes, both by empirical methods and with the help of mathematical methods, including modeling (Volterra, 1928; Bazykin, 2003; Riznichenko, 2017).

Finally, biogeochemical cycles, i. e., processes of dynamic exchange of chemicals between organisms from prokaryotes to higher animals and plants and elements of the biosphere (soil, water and air) can be classified as cyclic processes at the ecological level (Van Cappellen, 2003; Zavarzin, 2003, 2011; Struyf et al., 2009).

Cyclical processes in biology are investigated using experimental and theoretical methods. Mathematical modeling is one of the main methods for their investigation, particularly in finding areas of stationary, oscillatory, and possibly chaotic behavior (Romanovsky et al., 1975; Schnol, 1996; Becks, Arndt, 2013).

The first works devoted to oscillatory biochemical processes belong to Alfred Lotka (Lotka, 1910). Lotka described the dynamics of biochemical processes using systems of nonlinear ordinary differential equations. Around the same time, Vito Volterra, independently of Lotka, developed the same models, but in application to population-ecological problems. These models were later called the “Lotka–Volterra models”. Further study of oscillatory chemical processes led to the discovery of Belousov–Zhabotinsky type systems, in which oscillations occur not only in time but also in space, and, therefore, can be described not only by ordinary differential equations, but also by partial differential equations (Zhabotinsky, 1974; Field, Burger, 1988; Mushtakova, 1997; Shnol, 2009).

This article presents a review of classical mathematical models that describe oscillatory behavior in biological systems and gives illustrations of methods for finding such systems using enzymatic oscillatory systems as an example. The role of gene networks in modeling oscillatory molecular-genetic systems is discussed. The factors influencing the presence or absence of oscillatory behavior in various molecular-genetic systems are given.

## Classical models and methods for modeling oscillatory processes

Among the first mathematical approaches describing oscillatory processes are models that have already become classical in the field of mathematical biology (Riznichenko, 2002). In one of the papers devoted to the theory of periodic reactions, Lotka studied a chemical reaction of the form (1):

**Formula:**
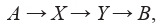
(1)

where X → Y is an autocatalytic process. Based on the law of mass action, Lotka described this reaction by the following differential equations (Lotka, 1910) (Formula 2):

**Formula:**
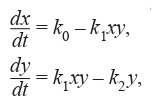
(2)

where k0, k1, k2 are the constant parameters and x, y are the concentrations of chemicals.

The following model, described by Lotka (Lotka, 1920) and then independently formulated by Volterra (Volterra, 1928), expresses two autocatalytic reactions (i. e. A → X and X → Y). The Lotka–Volterra model has the following form (3):

**Formula:**
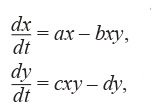
(3)

where a, b, c, d are the rates of transformation of some substances into others, x, y are the concentrations of chemicals. This model is also known as the “predator–prey system”, which is used in population dynamics to explain periodic fluctuations in the abundance of individuals in populations.

In the same period a paper with the van der Pol and van der Mark oscillator model was published (van der Pol, van der Mark, 1928). They modeled the heart as three connected relaxation systems: sinus, atrium and ventricle. As such a system, the authors chose a system consisting of a neon lamp, a condenser, a resistance, and a battery, which is capable of producing relaxation oscillations. However, this system simulates only some modes of heart operation due to the complexity of the object under study. The model is described by an equation of the form (4):

**Formula:**
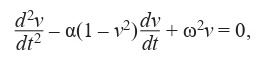
(4)

where α is a positive value, which is an oscillator parameter (responsible for non-linearity and damping of oscillations), ω – oscillation frequency, v – the value corresponding to the heart rhythm signal.

This model is noteworthy because it has found an application not only in biology problems, but also in physics and other sciences. For example, the review (Kuznetsov et al., 2014) presented a number of problems in which this oscillator was applied; in particular, the authors gave details on modeling human body processes, such as colonic myoelectric activity and processes of excitation and inhibition of neurons. In the paper (Rompala et al., 2007), the authors considered three van der Pol oscillators to study the in-phase mode, which corresponds to the synchronized periodic behavior of circadian rhythms. Moreover, two of them correspond to the eye models, and the third oscillator is a model of the brain (mainly represented by the pineal gland), through which the interconnection of the first two is performed. They considered the periodic change of melatonin concentration under the influence of circadian rhythms as a possible scheme of connection between the eyes and the pineal gland.

In 1965, an article by Brian Goodwin (Goodwin, 1965) was published, which raised the question of the oscillatory motion role in the organization of cellular processes over time. For the mathematical study of oscillatory behavior in model systems involving enzyme regulation processes, he introduced certain concepts of thermodynamic nature. In the article, the author cited a model of the process of genetic control of enzyme synthesis (Formula (5)):

**Formula:**
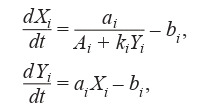
(5)

where Xi is an mRNA concentration of the i th species, Yi is a protein (repressor) concentration of the i th species, ki – parameter, which describes the interactions between the DNA and the repressor.

Another classic example is the Higgins model (Higgins, 1964) of oscillatory reactions in the glycolysis system, the scheme of which is shown below (Formula (6)):

**Formula:**
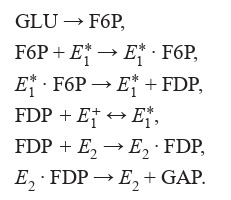
(6)

Here GLU, F6P, FDP, GAP are designations of biochemical substances that enter into reactions, E * 1 – the active form of the enzyme (phosphofructokinase), E + 1 – the inactive form of the enzyme, E2 – the enzyme that is a combination of aldolase and triose phosphate isomerase

Higgins considered general pathway types of enzymatic reactions in glycolysis in which the chemical mechanism exhibits oscillatory behavior. Therefore, in his work, he takes into account the following conditions: (1) one of the chemicals must activate its own production (assuming the concentration of the second substance is constant); (2) the second substance must tend to inactivate its own net production; (3) there must be a cross-coupling of the interaction of substances. If an increase in the first substance activates the production of the second substance, then an increase in the second substance inhibits the production of the first, and vice versa

Sel’kov in his classic article (Sel’kov, 1968), in accordance with the mass action law, gave a mathematical model of the glycolytic system based on the phosphofructokinase (PFK) transformations (Formula (7)):

**Formula:**
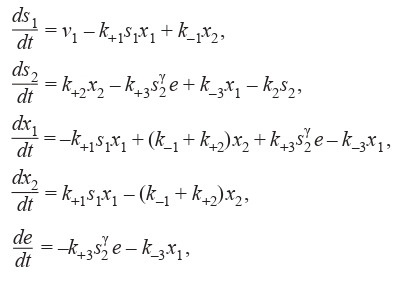
(7)

where s1 – the substrate (ATP), v1 – the inf low rate of the substrate from some source, s2 – the product (ADP), v2 = k2s2 – the outflow rate of the product from the system, e – free enzyme (phosphofructokinase), which is inactive on its own, but becomes active when combined with product molecules as a complex – ES γ 2 , x1 – the molecule of the complex (ES γ 2), x2 – the molecule of enzyme-substrate complex (S1ES γ 2 ), s γ 2 – product molecules that enter into a complex with the free enzyme, γ > 1 – the parameter responsible for the number of the product molecules, k+1, k+2, k+3 – rates of direct reactions, k–1, k–3 – rates of reverse reactions, t – time.

Goldbeter and Lefever (Goldbeter, Lefever, 1972) presented a model of the glycolytic system, which is a generalization of the models presented by Higgins (Higgins, 1964, 1967) and Sel’kov (Sel’kov, 1968). The model is based on the mechanism of positive feedback, namely, the activation of the product by the enzyme PFK.

In the article (Boiteux et al., 1975), the authors not only analyzed the allosteric model of the oscillatory reaction of phosphofructokinase, but also made experimental verification of theoretical predictions. The data obtained for the model agreed well with the experimental data.

In 2000, a model of a yeast population consisting of a small ensemble of individual cells was presented to describe the phenomenon of synchronization of glycolytic oscillations. In this case, the communication between the cells was performed through the exchange of acetaldehyde (Bier et al., 2000). Glycolytic oscillations were also studied using stochastic methods and chaos theory in (Bashkirtseva, Ryashko, 2017); Selkov’s minimal model was taken as the basis, and in the article (Ryashko, 2018) a two-dimensional Higgins model was used.

In biochemistry, the processes of changing the concentration of ions in cells, which can increase or decrease the activity of enzymes, participate in the metabolism of carbohydrates, lipids and proteins, as well as play an important role in signal transduction through signaling pathways and are responsible for cell excitability, are actively studied. One of such processes is periodic changes in calcium ion concentrations. Anumber of mathematical models have been developed to study these periodic processes. Amodel describing calcium ion concentration fluctuations was first proposed in (Dupont, Goldbeter, 1989) (Formula (8)):

**Formula:**
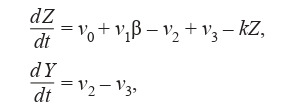
(8)

where Z – the cytosolic calcium concentration, Y – the calcium concentration in IP3 (inositol-1,4,5-triphosphate) endoplasmic reticulum, vi (i = 0, …, 3) – reaction rates.

They analyzed the conditions for the emergence of stable fluctuations based on the mechanism of calcium-induced calcium release (CICR). In a number of studies (Goldbeter et al., 1990; Dupont et al., 1991; Dupont, Goldbeter, 1993), the authors continued their researches of calcium concentration fluctuations based on the same minimal model.

At the same time, papers (Meyer, Stryer, 1988; Meyer, 1991) in which the authors investigated fluctuations in calcium concentrations by considering the mechanism of inositol cross-coupling (ICC) IP3 with extracellular, cytosolic, and endoplasmic Ca2+ have been coming out. Lavrentovich and Hemkin (Lavrentovich, Hemkin, 2008) proposed a model for spontaneous Ca2+ oscillations in astrocytes that takes into account the mechanisms presented above as well as IP3 production in a receptor-independent manner

After Goldbeter and Dupont had published their results, the authors of the article (Kraus et al., 1996) tested the hypothesis that in unexcited cells the amplitudes of oscillatory processes can be cell type-specific and vary with Ca2+ diffusion. They performed their study using stochastic computer modeling on a two-dimensional Ca2+ oscillation model.

Analysis of oscillatory processes in living systems shows that the most important factor in the emergence of cyclic behavior is a feedback in the system (Kolchanov et al., 2000). Adistinction is made between positive and negative feedbacks, which was once discussed by Goodwin, Walter, Cardon, Iberall and other researchers (Goodwin, 1965; Walter, 1969, 1970; Cardon, Iberall, 1970). Both types of these feedbacks can influence the emergence of cyclic dynamics in the system, as has been shown in works (Likhoshvai et al., 2001; Goldbeter, 2002; Tyson et al., 2003).

At the molecular level, the principle of feedback regulates a huge number of enzymatic reactions simultaneously going on in a living cell, the rate of which can be affected by such compounds as inhibitors, activators, cofactors, allosteric effectors, etc. As early as 1913 an article (Michaelis, Menten, 1913) by biochemists Michaelis and Menten was published, in which the scientists derived the equation for the dependence of the reaction rates catalyzed by the enzyme on the concentration of the substrate. Later, the researchers have showed that, using computational methods, optimizing the parameters of the equation by approximating the model data to the experimental data corresponds to the results, which were obtained manually by Michaelis and Menten for their constant.

Not long ago, a review was conducted of how methods for quantitative analysis of enzyme kinetics have emerged, changed, and been modified over a century (Johnson, 2013). In the same year an article (Goldbeter, 2013) examined the influence of Michaelis–Menten kinetics on oscillatory behavior in enzymatic systems, namely, in glycolysis from phosphofructokinase activity and in the cell cycle from cyclindependent kinases

Novák and Tyson reviewed examples of oscillatory processes and formulated the necessary conditions for oscillations in the system: negative feedback, time delay, sufficient ‘nonlinearity’ of the reaction kinetics and proper balancing of the timescales of opposing chemical reactions (Novák, Tyson, 2008).

In a recent review (Tyson et al., 2019), the authors compiled various approaches to modeling the dynamics of the behavior of biochemical regulatory networks that have been developed over the past 50 years. Models such as Boolean (logical) ones, models consisting of piecewise-linear or fully nonlinear ordinary differential equations, and stochastic models (including hybrid deterministic/stochastic approaches) are considered. The authors focused on two approaches: modeling genetic control systems as networks of Boolean switches and metabolic and signaling networks using systems of nonlinear ordinary differential equations. They considered only spatially homogeneous systems. The authors showed the advantages and disadvantages of each method depending on the type and amount of available experimental information.

The models, which we reviewed in this section, are summarized in Table 1.

**Table 1.**
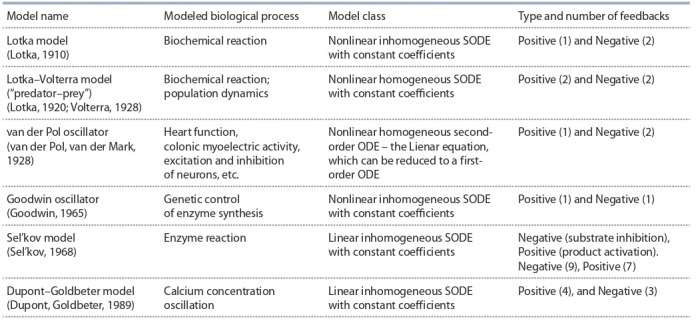
Brief characteristics of a number of classical models with oscillatory behavior Notе. ODE – ordinary differential equation, SODE – system of ODEs.

## Application of gene networks in the modeling of oscillatory systems

Modeling of metabolism is often associated with modeling of genetic regulation (Smolen et al., 2000; Hecker et al., 2009). The concept of gene networks plays an integrative role in this case (Kolchanov et al., 2013; Ocone et al., 2013).

The main task of the theory of gene networks is to identify causal relationships between the structural and functional organization of gene networks and their dynamic properties (Chen et al., 2010; Kolchanov et al., 2013). The structural and functional organization of gene networks is understood as a set of molecular-genetic and biochemical processes, while the dynamic properties are understood as the kinetics of changes in the concentrations of end products over time.

Computer analysis and modeling of small gene networks, especially hypothetical gene networks, provides very valuable information for understanding the fundamental features of the dynamics of regulatory gene networks. Likhoshvai and his colleagues developed a theory linking the structural and functional organization of hypothetical gene networks with their dynamics (Likhoshvai et al., 2001, 2003, 2004; Fadeev, Likhoshvai, 2003; Demidenko et al., 2004). Namely, the concept of a hypothetical gene network was defined; rules for formalizing the description and assembling mathematical models from them are given. The (n, k)-criterion for predicting some properties of the models by the structure of the network graph is formulated; 4 classes of the hypothetical gene network are introduced according to the types of regulatory links in the network; and analytical and numerical studies of the models for each class of the hypothetical gene network are given.

**Fig. 1.**
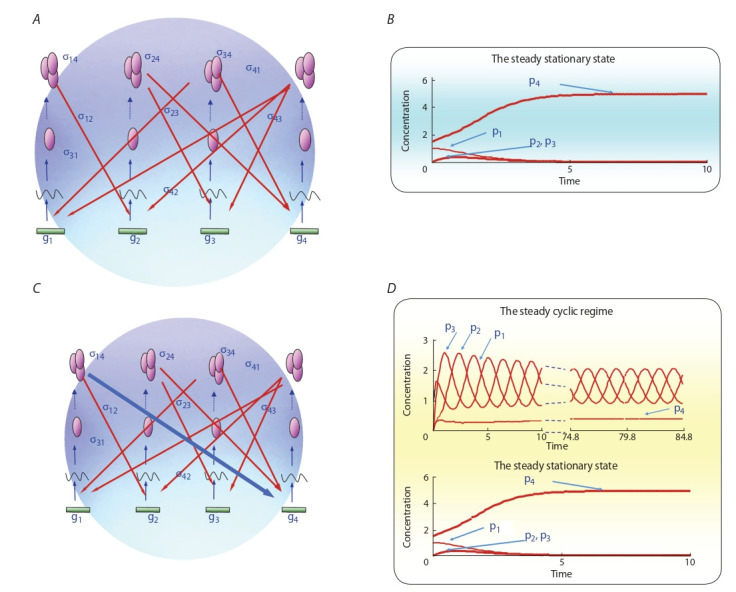
Relationship between the structural model (graph) of the hypothetical gene network and its dynamics: A, structure of the hypothetical gene network of 4 genes and 8 negative feedbacks; B, dynamics of the hypothetical gene network A; C, structure of the modified gene network A – to which an additional negative regulatory link was added – inhibition of gene g4 expression by the product of gene g1 (marked by a blue arrow); D, dynamics of the modified gene network В. Here, green rectangles (gi ) are genes, broken line is RNA corresponding to a certain gene, pink ellipse is polypeptide chain of protein, several pink ellipses are a complex of proteins performing gene regulation (regulation is shown by a red arrow). Modified according to (Kolchanov et al., 2008).

In particular, it was first theoretically and then numerically demonstrated how the appearance of a new regulatory link leads to a qualitative change in the dynamics of the gene network (Fig. 1). Thus, the addition of another regulatory link in the gene network cardinally changes the possible modes of functioning of this network – if only one stationary state was possible in the initial network, then after adding another regulatory link, there are already two possible states – stationary (as in the previous case) and cyclic mode.

The connection between the structures of gene networks and the presence of dynamic cycles in them has been studied for many years. In particular, the connection between network structure and cyclic dynamics has been theoretically shown (Likhoshvai et al., 2003; Demidenko et al., 2004; Novák, Tyson, 2008). Elowitz and Leibler designed and studied a genetic network of a repressilator, in which the network under study is locked into a cycle of interactions based on the principle of negative feedbacks. The authors experimentally showed that this type of network has an oscillatory mode of behavior (Elowitz, Leibler, 2000).

In the Sobolev Institute of Mathematics SB RAS is studied the qualitative theory of dynamical systems describing various gene networks that are regulated by feedbacks. Golubyatnikov and his colleagues have studied in their works (Gaidov, Golubyatnikov, 2007; Golubyatnikov et al., 2010; Akinshin, Golubyatnikov, 2012; Golubyatnikov, Kazantsev, 2016; Golubyatnikov, Kirillova, 2018) the existence and uniqueness of periodic solutions, existence of closed trajectories, cycle stability, etc. in such systems. The interest in the analysis of the behavior of such trajectories is to correspond them to the modes of functioning of gene networks. An article (Likhoshvai et al., 2020) showed that oscillatory trajectories are present in models of the simplest circular gene networks and they are stable.

## The method for finding oscillating molecular-genetic systems

**Fig. 2.**
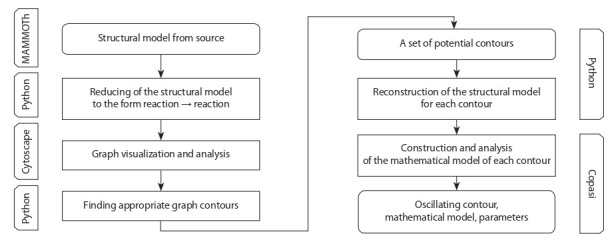
Scheme of the algorithm for searching oscillatory enzymatic systems.

In this paragraph, we describe the algorithm for searching the oscillatory molecular-genetic systems (the algorithm scheme is shown in Fig. 2). It uses information resources both developed by the authors and widely known in systems biology. In particular, the MAMMOTh database is a source of structural and mathematical models of Escherichia coli metabolic reactions (Kazantsev et al., 2018). Cytoscape (cytoscape.org) is a tool for working with structural models and Copasi (Hoops et al., 2006) is a tool for reconstructing and investigating mathematical models. Python (python.org) is both a data processing tool and a link between the steps.

The input of the algorithm takes a structural model – a gene network graph with typing of model elements and their relations. There are two types of nodes in the graph: biological substances (molecules and their groups) and processes (or reactions). The edges specify the following relations between the nodes: substance is a substrate in a reaction, substance is a product of a reaction, and substance is a regulator of a reaction. This information can be obtained directly from models in SBML (Hucka et al., 2003), SBGN (Le Novère et al., 2009), from other tools for building structural models, or from Python scripts. To date, any database that has information on metabolic pathways and molecular-genetic systems models can be used as a data source. The best-known databases are KEGG (Kanehisa, Goto, 2000), GeneNet (Ananko, 2002), MetaCyc (Caspi et al., 2016), EcoCyc (Keseler et al., 2017), BioModels (Le Novere et al., 2006; Malik-Sheriff et al., 2019), etc

In this article, we considered a special case of moleculargenetic systems – the oscillatory enzymatic systems. Analysis of the literature (Likhoshvai et al., 2001; Novák, Tyson, 2008; Tyson, Novák, 2010; Wong, Huck, 2017) allows us to identify the following key characteristics of potentially oscillating contours: (1) the closure of the contour (oriented path from node A to it, through N nodes, where N > 3); (2) the orientation of the contour in one direction, with the last node having an edge of regulatory inhibitory influence on the first node in the contour (as in the contour in Fig. 4, a, for example).

**Fig. 3.**
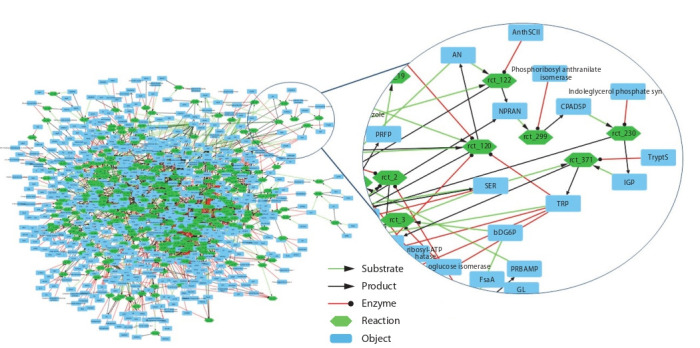
Structural model (graph G) constructed from 300 subsystems of E. coli metabolic pathways taken from the MAMMOTh database (Kazantsev et al., 2018). Here and in the Fig. 4 the following notation is used: Blue squares represent substances involved in metabolic reactions. Green hexagons indicate reactions, with arrows in/out specifying the relations of the interacting substances: green arrows specify the reaction substrates; black arrows specify the reaction products; red arrows specify the regulatory effects of the substances on the reactions.

A graph of 300 subsystems (Fig. 3) representing models of E. coli metabolic reactions taken from the MAMMOTh database was taken as initial data

**Fig. 4.**
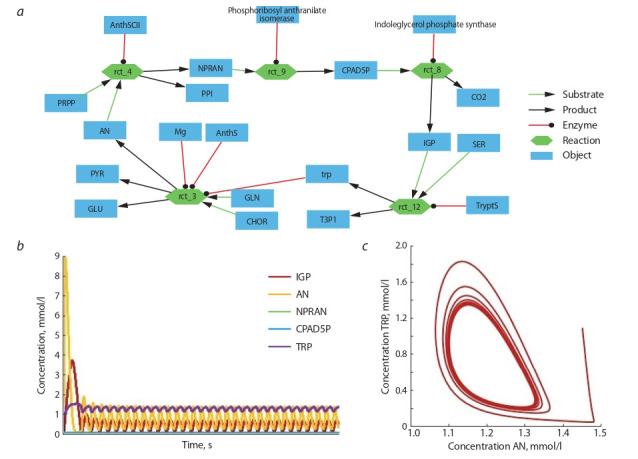
Potentially oscillating contour and its numeric analysis. а, studied contour that is a part of the metabolic pathway of tryptophan biosynthesis; b, the plot with the results of the simulation, the dependence of the concentration of the specified substances on time; c, phase trajectory plot based on simulation results, where the abscissa and ordinate axes are the concentrations of anthranilate (AN) and L-tryptophan (TRP), respectively

The construction of a mathematical model of a potentially oscillating contour can be performed both in general-purpose engineering simulation environments (Matlab, Mathematica or Scilab) or in specialized environments designed for the simulation of molecular-genetic systems (Copasi, CellDesigner (Funahashi et al., 2003), VCELL (Schaff et al., 1997; Cowan et al., 2012), etc.). The advantage of the latter is the ready library of tools for reconstruction, computational experiments and model analysis.

Six potentially oscillating contours were found in the analyzed graph, and during the numerical analysis of the reconstructed mathematical model oscillatory behavior was shown for only one of them (Fig. 4). The mathematical model of the contour was constructed based on the reactions related to the metabolic pathway of tryptophan biosynthesis (Formula (9)):

**Formula:**
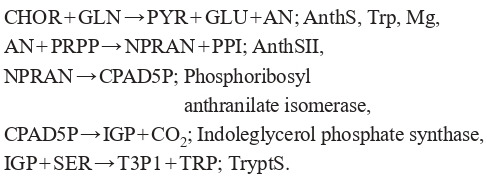
(9)

Here CHOR, GLN, PYR, GLU, AN, PRPP, NPRAN, PPI, CPAD5P, IGP, SER, T3P1, TRP – before the semicolon are the designations of the biochemical substances involved in the reaction, and after that are regulators of reactions. Full names of substances are given in Table 2.

**Table 2.**
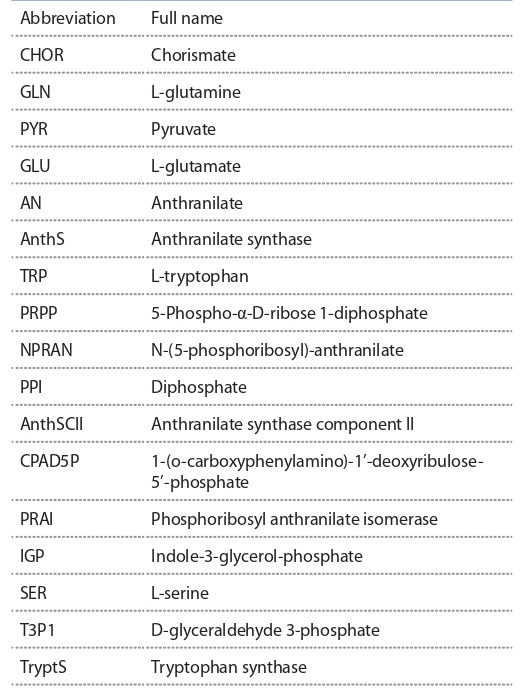
List of full names of biochemical substances used in the model

The model was built in Copasi and consists of 5 differential equations. (Formula (10))

**Formula:**
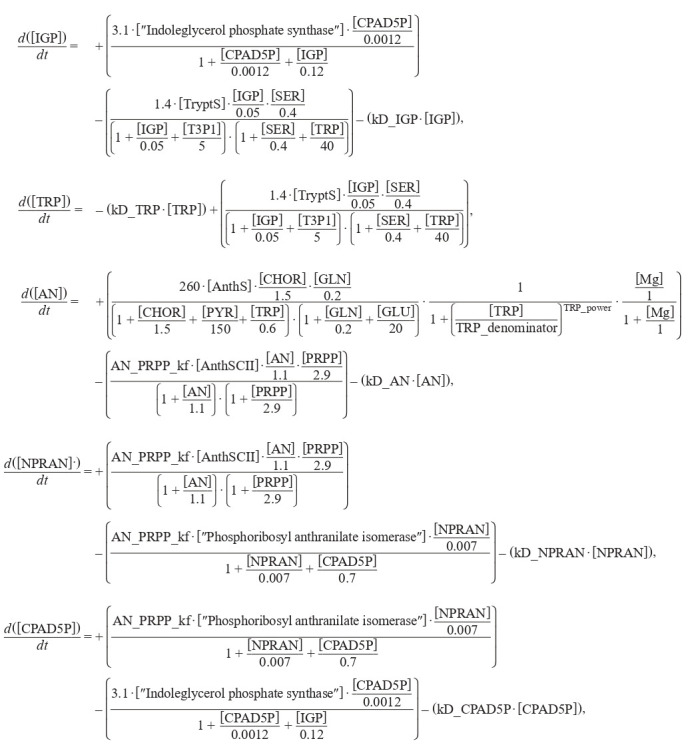
(10)

where kD_“substance name” are degradation constants of corresponding substances, parameters TRP_power and TRP_denominator varied in the process of searching for oscillatory behavior of the system. The given numerical parameters were taken from the MAMMOTh database.

The mathematical model of only one of the six contours found exhibits with oscillatory behavior. As we considered, a network consisting of only 300 enzymatic reactions, which had mathematical models adapted to the experimental data, may explain such a small number of contours. In turn, there are currently not many such mathematical models for describing the enzymatic reactions of biological systems. Thousands of existing models presented in databases are often automatically generated, as in the Path2Models project for the biomodels.net database, for example. Experimentally measured kinetic parameters of biochemical reactions are becoming increasingly scarce. Using graphs with higher dimensionality (full-genome models) to study oscillatory behavior will increase the number of variants to be tested, but this will require additional consideration of the regulatory component of genetic synthesis. All of these things present additional challenges in the study of this problem.

## Conclusion

The article gives an overview of a number of biological processes of oscillatory nature, as well as mathematical models of these processes. It is noted that the most important factor for the emergence of cyclic behavior is feedbacks in the system. Based on the analysis of these factors, an algorithm for finding cyclic modes of functioning of molecular-biological systems is given.

## Conflict of interest

The authors declare no conflict of interest.
